# Does Perceived Nuisance Abundance of Water Plants Match with Willingness-to-Pay for Removal? Contrasts Among Different User Categories

**DOI:** 10.1007/s00267-024-02046-5

**Published:** 2024-09-18

**Authors:** Jan E. Vermaat, Kirstine Thiemer, Bart Immerzeel, Susanne C. Schneider, Keneilwe Sebola, Julie Coetzee, Antonella Petruzzella, Samuel N. Motitsoe, Mathieu Baldo, Benjamin Misteli, Gabrielle Thiébaut, Sabine Hilt, Jan Köhler, Sarah Faye Harpenslager

**Affiliations:** 1 Faculty of Environmental Sciences and Natural Resource Management, Norwegian University of Life Sciences, P.O. Box 5003, 1430 Ås, Norway; 2 Norwegian Institute for Water Research, Økernveien 94, 0579 Oslo, Norway; 3 Norwegian Institute for Nature Research, Sognsveien 68, 0855 Oslo, Norway; 4 Centre for Biological Control (CBC), Department of Botany, Rhodes University, PO Box 94, Makhanda (Grahamstown), 6140 South Africa; 5 Centre for Biological Control (CBC), Department of Zoology and Entomology, Rhodes University, PO Box 94, Makhanda (Grahamstown), 6140 South Africa; 6 School of Animal, Plant and Environmental Sciences, University of the Witwatersrand, Private Bag 3, Johannesburg, South Africa; 7 Université de Rennes, UMR 6553 CNRS ECOBIO, 263 Avenue du Général Leclerc, Campus Beaulieu, 35042 Rennes, France; 8 WasserCluster Lunz, Dr. Carl Kupelwieser Promenade 5, A-3293 Lunz am See, Austria; 9 Dept. of Community and Ecosystem Ecology, Leibniz Institute of Freshwater Ecology and Inland Fisheries, Müggelseedamm 301, 12587 Berlin, Germany; 10 B-Ware Research Centre, Postbus 6558, 6503 GB Nijmegen, The Netherlands; 11 Present Address: Department of Ecoscience, Aarhus University, Roskilde, Denmark; 12 Present Address: Dept. of Community and Ecosystem Ecology, Leibniz Institute of Freshwater Ecology and Inland Fisheries, Müggelseedamm 301, 12587 Berlin, Germany

**Keywords:** macrophytes, mass development, integrated weed management, questionnaire survey

## Abstract

Dense beds of water plants can be perceived as nuisance, but this perception, however, may not be similar for different user categories, and this may affect their willingness-to-pay (WTP) for plant removal. A questionnaire survey was used to test this for residents and visitors and find underlying socio-cultural or economic drivers. We studied five cases where nuisance water plant growth is managed: the rivers Otra (Norway) and Spree (Germany), and the lakes Kemnade (Germany), Grand-Lieu (France), and Hartbeespoort Dam (South Africa). We used a different payment vehicle for residents (annual household tax) and visitors (tourist tax). The survey included questions on days spent on specific types of activity per year, the importance attached to different functions and activities, overall environmental attitude, perception of the plants, socio-demographic respondent characteristics and WTP for increased plant removal. We observed no increase in WTP for increased removal in most sites. The two most important drivers of variation in current WTP were income, and whether respondents were engaged in boating and angling and thus perceived the plants negatively. Variation in WTP among sites was considerable, and mainly related to the mixture of activities among respondents. Differences between residents and visitors were less important than those among sites. Our observations bear importance for water management: information on differences in experienced nuisance among user categories and the frequency of use by these categories is useful as guidance for the design and implementation of any plant removal plan.

## Introduction

Negative effects of recreation on littoral and submerged vegetation are well documented (Murphy and Eaton 1983, Hadwen et al. 2003, Ostendorp et al. 2009, Venohr et al. 2018, Nikolaus et al. 2020, Schafft et al. 2021, Wegner et al. 2023). Aquatic vegetation may, however, also be perceived as too dense and hindering recreative activities. This perception is activity- and context-dependent (Clayton 1996, Villamagna and Murphy 2010, Verhofstad and Bakker 2019, Thiemer et al. 2023). Verhofstad and Bakker (2019), for example, suggest nuisance cover threshold levels for submerged plants to be 5%, 10% and 50% for boating, swimming and angling, respectively. The local importance of different recreational activities, however, should inform longer-term management strategies (Verhofstad and Bakker, 2019) as well as decisions on short-term measures (Thiemer et al., 2023). Experienced nuisance has led to a range of control and removal measures, including the use of herbicides, herbivorous grass carp or host-specific insects, sediment coverage and mechanical harvesting, where the latter is the main approach worldwide (Pieterse and Murphy 1990, Hussner et al. 2017, Hill and Coetzee 2017, Thiemer et al. 2021). Water plant removal can have considerable costs (Hilt et al. 2006; Schneider unpublished: median 58, range 4–224 € ha^−1^ y^−1^ for our study sites) and it generally has to be repeated regularly (Pieterse and Murphy, 1990). In some cases, the cost of removal can be considerably lower than the financial benefits (e.g., Arp et al. 2017), but estimating cost recovery is not straightforward (Podraza et al. 2008, Boerema et al. 2014). These costs may be borne in various ways, e.g. by public water authorities that are paid from different forms of taxes including local tourist tax and access fees, by riparian private landowners, or a combination of these. Also, benefits may be distributed unequally over different user groups (Bergstrom et al. 1996, Boerema et al. 2014). This raises the question whether specific groups of users would be willing to pay more for nuisance plant removal, and whether this willingness would be a function of removal intensity. Additionally, there may be differences in the willingness to pay (WTP) between residents and visitors that are less familiar with the local situation. Better estimates of the societal demand for aquatic plant removal, linked to user characteristics as well as ecosystem characteristics, can help to design targeted cost recovery mechanisms and provide budgets for the management of mass development of aquatic plants.

The literature on patterns in appreciation of and willingness-to-pay (WTP) for aquatic plant removal is limited (Bergstrom et al. 1996, Beardmore 2015, Navrud 2015, Dehez 2023). Using latent class modelling, Beardmore (2015) found that different groups of boaters in Wisconsin (USA) had different concerns and priorities, with anglers being concerned about fishing quality and sightseers with lakeshore development and the presence of apparent natural habitat. An increased abundance of aquatic plants was not among the higher-ranking issues. His results support that appreciative, non-consumptive activities are more strongly related to environmental concern than consumptive activities while he did not find unequivocal support for the hypothesis originally proposed by Dunlap and Heffernan (1975) that environmental concern increases with increasing participation in outdoor recreation. Bergstrom et al. (1996) studied spending patterns and regional economic effects with an input-output model and a preference survey among different groups of recreational users of a large reservoir in Alabama (USA) for different scenarios of surface plant cover. The number of visits by anglers, other categories of visitors and total expenditures to the local economy by non-local visitors were highest at 30% plant cover. Dehez (2023) carried out a survey addressing different forms of willingness to participate in controlling mass development of invasive aquatic plants, including behavioral change, assistance in manual removal, and payment. Respondents engaged in different forms of recreation preferred different forms of participation. The literature thus suggests that there may be differences in perceived nuisance among different categories of recreative users leading to differences in WTP for plant removal programs, but there is little basis to further generalize on the importance of geographical, economical or socio-cultural aspects of context.

Here, we investigate patterns in levels of WTP for water plant removal among resident and visitor user groups and explore potential explanatory factors. We use data of a survey that was carried out in five different case study areas experiencing nuisance mass development of aquatic plants as part of a larger project (Thiemer et al. 2023, Vermaat et al. 2024). Specifically, we investigated whether 1) respondents are willing to pay more for increased aquatic plant removal, 2) different types of recreative users differ in their WTP for aquatic plant removal, and if this is related to their perceived nuisance and 3) whether underlying socio-cultural or economic drivers for the observed patterns in stated WTP for plant removal can be identified.

## Materials and Methods

### Study Sites

The five study sites were selected based on reported major aquatic plant nuisance problems (Table 1; Misteli et al. 2023). We included rivers and lakes that contrast in plant growth form and have different predominant types of use and geographic setting. One of the sites is a nature reserve (Lake Grand-Lieu) with restricted access to the core area but considerable recreational use around the margins, whereas all other sites are freely accessible (Table 1; see also Thiemer et al. 2023).

**Table 1:** Description of the study sites (adopted from Thiemer, 2022)

Site (country), coordinates (lat/long)^a^	Area, annual mean discharge	Important current forms of use	Gross median monthly income (PPP2019€) national-our sample	Nutrient status of the water	Nuisance species	Mean plant biomass (g DW m^−2^)
River Otra at Rysstad (Norway) 59.088/−7.550	Upstream of the study reach 11 km length and 210 ha, 69 m^3^ s^−1^	Hydropower, recreation	3800–750	oligotrophic	Submerged *Juncus bulbosus* (bulbous rush) canopy often reaching the water surface	148 ± 35
River Spree from Grosse Tränke to Lake Dämeritz (Germany) 52.430/−13.678	reach of 34 km length and an area including floodplain of 2050 ha, 14 m^3^ s^−1^	Recreation, agriculture in the floodplain	2500–3500	eutrophic	Submerged and emergent *Sagittaria sagittifolia* (arrowhead)	335 ± 61
Lake Kemnade (Germany) 51.416/−7.260	125 ha, created in the valley of the river Ruhr	Recreation, hydropower, drinking water, flood regulation	2500–2750	eutrophic	Submerged *Elodea nuttallii, (*Nuttall’s waterweed*)* canopy reaching the water surface	421 ± 180
Lake Grand-Lieu (France) 47.133/−1.674	Seasonal variation with summer drawdown, 3500 – 6300 ha; summer level open water 2700 ha and wet pastures 2400 ha	Strict nature reserve, professional fishermen; recreation and agriculture along its banks	2333–1750	eutrophic	Emergent and amphibious *Ludwigia grandiflora* and *L. peploides* (water primrose) mixture of two species difficult to separate for the non-expert	183 ± 85
Lake Hartbeespoort Dam (Republic of South Africa) −25.749/ −27.833	Reservoir, 1850 ha	Irrigation, drinking water, recreation	391–2750	hypertrophic	Free-floating *Pontederia crassipes* (water hyacinth)	972 ± 137

^a^Negative latitudes are S of the equator, negative longitudes are E of Greenwich

### Questionnaire Design

**Fig. 1:**
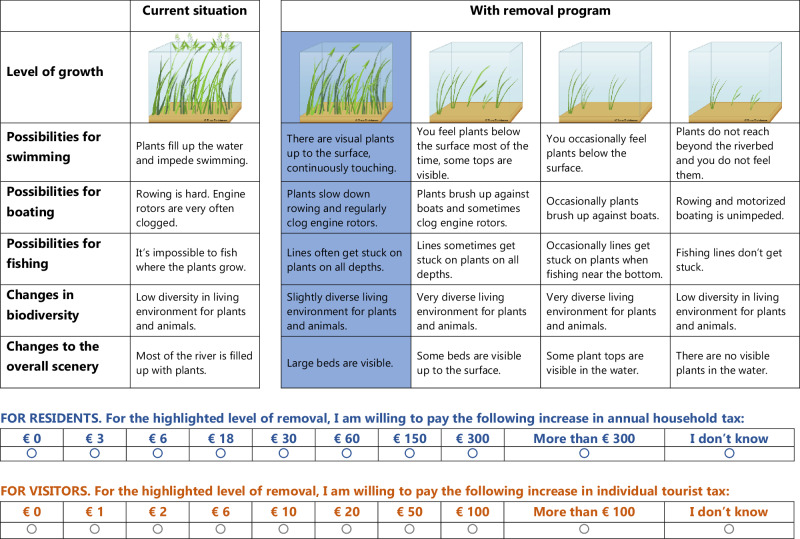
Excerpt from the willingness-to-pay question in the survey for the German case of the River Spree. Presented are the different levels of removal intensity, the consequences for different uses and the ecosystem, and the different levels of payment. Here ‘level 1’ of four is highlighted. For a complete survey refer to Thiemer et al. (2023)

The questionnaire consisted of a common set of 28 questions in all five sites, with types of activity and levels for willingness-to-pay adjusted to the local situation. Briefly, questions covered whether plant growth was experienced as nuisance, type of activities engaged in, importance attached to different types of use of the lake or river, WTP for various levels of plant removal, and a set of socio-economic queries also including a standardized set of 15 New Environmental Paradigm questions assessing an overall environmental mindedness of a respondent (the NEP scale, Dunlap et al. 2000). We had separate parts for residents and visitors. A complete example is supplementary material to Thiemer et al. (2023). Questionnaires were translated into the local language by native speakers and pre-tested among a group of colleagues and students in each country. Questionnaires were offered on-site in a printed version by our local co-authors with the help of local assistants, and digitally via a QR code presented on flyers and in local media, via a link sent by e-mail to local interested groups (e.g. boating and angling communities) or posted on social media. The questionnaires were filled in anonymously and complied with the data protection and privacy rule and regulation in the respective countries. The payment vehicle used for residents was an increase in annual household tax, whereas we chose an individual tourist tax for visitors. Respondents were confronted with four successive panels showing levels of increasing aquatic plant removal and four to five characteristics of the study site, e.g., possibility for boating, changes in biodiversity, changes in the scenery. Respondents were then asked to choose the payment level they would be willing to pay for each of the four removal intensities (Fig. 1). Nine payment levels were chosen with 0.1% of the purchasing-power-parity-adjusted median annual income as midpoint of the range for residents following the recommendation of Johnston et al. (2017). The different levels span a range from zero to 10 times this midpoint and include a ‘more than this’ last option as well as an ‘I do not know’ option. For the three sites in the euro-zone we used the same WTP-range (low end – midpoint – high end): 0 – 30 – 300 € for residents and 0 – 10 – 100 € for visitors. Similar, but plausibly rounded classes in local currencies were used in Norway and South Africa. Currency conversion rates of December 2019 were used. Together, this provided a realistic and plausible WTP range (Johnston et al. 2017), which allows for comparability among the five sites. Pre-testing of the survey is described in Thiemer et al. (2023). The surveys were done in January 2020 (South Africa) and June to September 2020 in the other sites. The latter period coincided partly with covid-19 lock-downs.

The compiled data set contained a total of 52 variables including 3 respondent descriptor variables for a total of 1780 respondents, not all of which had completed all questions (Table 2). The total number of completed answers depended on the question and ranged from a low 646 (respondents revealing their gross monthly income) and 737 (respondents answering to be engaged in swimming) to a high 1328 (respondent answering the benefit ‘importance of clean water for nature’). The WTP questions were answered by 477 visitors and 662 residents.

**Table 2:** Summary overview of the responses given to the questions included in the presented analyses.

Variable	Mean ± SE (N, min – max)	p (site)	p (visitor-resident)	p (interaction)
Days spent on the following activities during the last 12 months:				
Days walking	22 ± 2 (1095, 0–365)	*<0.001	*<0.001	*<0.001
Days running	7 ± 1 (1113, 0–365)	0.205	*<0.001	0.153
Days cycling	12 ± 1 (1111, 0–365)	0.051	*<0.001	0.841
Days orienteering	0.1 ± 0.1 (1119, 0–50)	*0.044	0.553	0.745
Days enjoying the view	39 ± 3 (1090, 0–365)	*<0.001	*<0.001	*0.001
Days motorized boating	4 ± 1 (1114, 0–280)	*<0.001	*<0.001	*0.001
Days rowing, canoeing or sailing	7 ± 1 (1207, 0–365)	*<0.001	*0.003	*0.006
Days angling	4 ± 1 (1108, 0–260)	*<0.001	*<0.001	*0.005
Days swimming	7 ± 1 (1047, 0–365)	*0.018	*<0.001	*0.036
Days just relaxing	18 ± 2 (1193, 0–365)	*<0.001	*<0.001	*0.001
Ranking the importance of a range of benefits to society (Likert scale 1–5, from very unimportant to very important):				
Provision of drinking water	4.2 ± 0.1 (1319)	*<0.001	0.117	*0.043
Possibilities for recreational angling	3.2 ± 0.1 (1319)	*<0.001	*0.001	*<0.001
Clean water for nature	4.5 ± 0.1 (1328)	*<0.001	*0.002	0.012
Habitat for plants and animals	4.5 ± 0.1 (1323)	*<0.001	*0.002	*<0.001
Swimming possibilities	3.8 ± 0.1 (703)	*<0.001	*<0.001	0.055
The fact that there is nature	4.5 ± 0.1 (1319)	*<0.001	*0.025	*0.007
Educational possibilities	3.9 ± 0.1 (1307)	*<0.001	0.126	*<0.001
Boating possibilities	3.8 ± 0.1 (995)	*0.039	*0.019	0.099
The beauty of the landscape	4.4 ± 0.1 (1316)	*<0.001	*<0.001	*<0.001
Water storage to prevent floods	4.2 ± 0.1 (1321)	0.068	*<0.001	0.307
Possibilities for water sports	3.9 ± 0.1 (1004)	*0.035	0.076	0.400
Water for irrigation	3.7 ± 0.1 (1136)	*<0.001	0.665	*0.002
Personal perception of the current presence of water plants (Likert 1–5, from very negative to very positive)	2.6 ± 0.1 (1297)	*<0.001	*<0.001	*<0.001
Do you find the current plant growth a nuisance (1 = no)	0.14 ± 0.1 (1328)	*<0.001	*0.050	*<0.001
Distribution of 100 points over the following five characteristics that affected the answer to the willingness to pay question (0–100):				
Points for swimming	18 ± 1 (931)	*<0.001	0.233	*<0.001
Points for boating	21 ± 1 (1237)	*<0.001	*<0.001	*<0.001
Points for angling	13 ± 1 (1236)	*<0.001	*0.012	*<0.001
Points for biodiversity conservation	32 ± 1 (1236)	*<0.001	*<0.001	0.061
Points for esthetics of the scenery	22 ± 1 (1237)	*<0.001	0.512	0.442
Travel distance (one way, km)	42 ± 3 (968, 0–8000)	*<0.001	*<0.001	*<0.001
Currently employed (1 = yes)	0.65 ± 0.1 (1224)	*<0.001	0.075	0.794
completed higher education (1 = yes)	0.54 ± 0.1 (1220)	*<0.001	0.109	*0.025
Currently living in a rural setting (1 = yes)	0.45 ± 0.1 (1222)	*<0.001	*<0.001	*<0.001
Gender (fraction male)	0.56 ± 0.01 (1334)	*0.035	0.128	0.611
Age	47 ± 1 (1200, 11 – 92)	0.356	0.250	*0.009
Gross monthly income (converted to euro)	2652 ± 77 (1107, 0–12000)	*<0.001	0.111	0.125
Mean NEP^a^ score over 15 NEP questions (Likert 1–5)	3.32 ± 0.01 (1190, 1.0–5.0)	*<0.001	0.040	0.189
WTP (willingness-to-pay) visitors (euro)^b^	7.6 ± 0.7 (476, 0–500) median= 2, 235 answered ‘0’	*<0.001	–	–
WTP residents (euro)^b^	22.8 ± 2.4 (663, 0 – 500) median = 5, 387 answered ‘0’	*<0.001	–	–
Confidence in the WTP question (Likert 1–5, very uncertain – very certain)	3.1 ± 0.1 (1247, 0–5)	*<0.001	0.106	0.276

Presented are overall means pooled across the five sites and across residents and visitors plus minus 1 standard error, the number of answers as well as minimum and maximum, also for the ordinal five-point Likert scores. For questions where the answer is yes, the fraction that answers ‘yes’ is indicated plus minus one standard error and the number of answers. The last three columns give the level of significance (*<0.05) in a two-way ANOVA for the contrast among sites, between visitors and residents, and their interaction

^a^The New Environmental Paradigm-score is considered an indicator of the strength of the environmental orientation of a respondent (from Dunlap et al. 2000; see also Immerzeel et al. 2022 and Thiemer et al. 2023)

^b^Level of significance for the willingness to pay results only from a one-way ANOVA among the five sites. Overall, the WTP of residents is significantly higher than that of visitors (Welch’s t-test, *p* < 0.001)

### Statistical analyses

**Fig. 2:**
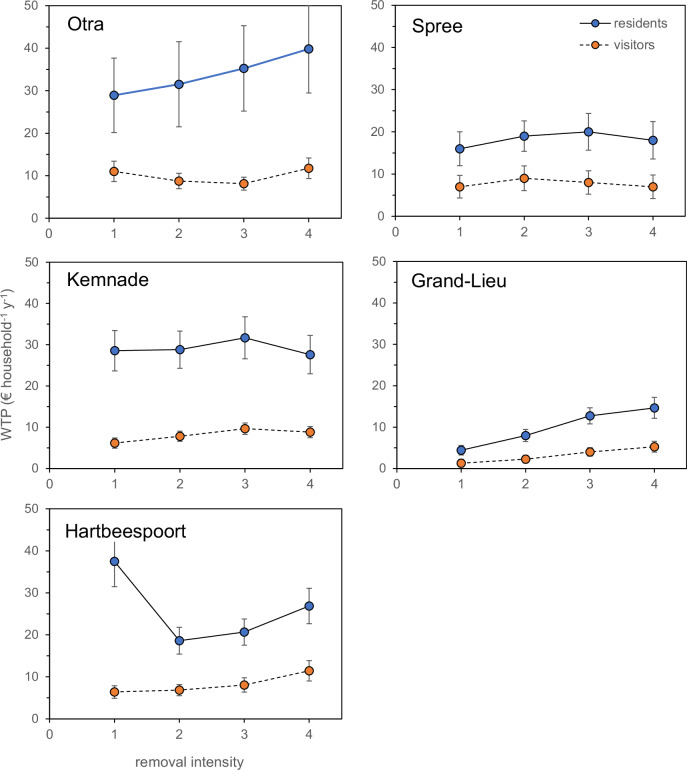
Willingness-to-pay (WTP) for increased levels of aquatic plant removal (cf Fig. 1) among residents and visitors of five different sites with perceived nuisance plant growth. Regressions of WTP against removal intensity were significant for Otra-residents and Grand-Lieu both categories (p ≤ 0.001, r^2^ > 0.98), the others were not. Presented are means ± 1 standard error

First, for each site the mean willingness-to-pay (WTP) was calculated separately for residents and visitors and regressed against removal intensity. Since the payment vehicle differs for residents and visitors and one respondent can only be either visitor or resident, the analyses had to be run separately, though the patterns can be compared. Second, contrasts among residents and visitors could be compared for a wide range of responses (e.g., environmental mindedness, perception of nuisance, numbers of days for different recreation activities) when the WTP-answers were excluded. This was done using two-way ANOVAs comparing among sites and between visitors and residents. Third, our analysis of WTP versus removal intensity most often showed no increase in WTP for increased removal and removal intensity often had no significant effect on WTP (Fig. 2). Therefore, for our exploration of potential explanatory underlying factors, the WTP for the moderate removal intensity level ‘2’ (see Fig. 2) was chosen. WTP for removal at level ‘2’ correlated well with that at level ‘3’ (r^2^ = 0.75, p < 0.001, slope = 0.84 ± 0.05, intercept 9 ± 27, 118 observation pairs). Covariance of this WTP with a wide range of potentially explanatory variables was explored with principal components analysis (PCA). Finally, analyses of co-variance were carried out testing the effect of site or user category as random factor and a range of covariates on WTP for this removal intensity level ‘2’ with. The number of covariates included was a compromise between power (less degrees of freedom mean lower power) and our interest in underlying explanatory trends (include more variables). For these ANCOVAs, respondents were allocated as ‘dedicated’ to a category if they reported to have spent more or equal to the average number of days on that activity. To illustrate this, respondents spent on average 1 day angling in Lake Kemnade (range 0–130, *N* = 317), and all spending at least 1 day were considered ’dedicated’. We compiled the following seven categories from the responses to question 6 in the survey (see Table 2): (1) those engaged mainly in walking, running or cycling, (2) rowing or canoeing or sailing, (3) angling, (4) using a motorboat, (5) swimming, (6) enjoying the view, or (7) just relaxing. It must be noted that attribution to one of these different categories is not exclusive, i.e., one can ‘just relax’, ‘enjoy the view’ and be ‘using a motorboat’ and report for these three categories the same or a different number of days. All analyses were performed in JAMOVI version 2.3.18, a spreadsheet interface using R (Navarro and Foxcroft, 2022).

## Results

### Willingness-to-pay for different removal intensities

We found little consistency in the WTP for increased plant removal intensity (Fig. 2): in only three out of ten cases we found a significant increase of WTP with removal: Otra-residents, Grand-Lieu residents and Grand-Lieu visitors. In Hartbeespoort Dam, residents were willing to pay considerably more for a low level of removal than for the subsequent, higher levels. This special pattern also contributed to our selection of the moderate removal level 2 (see Methods) for further analysis of potential underlying patterns. Overall, with a substantial overlap in range of WTP levels in their respective payment vehicles, residents had a significantly higher WTP than visitors (Table 2, Fig. 2).

### Differences in willingness-to-pay among recreational users

**Fig. 3:**
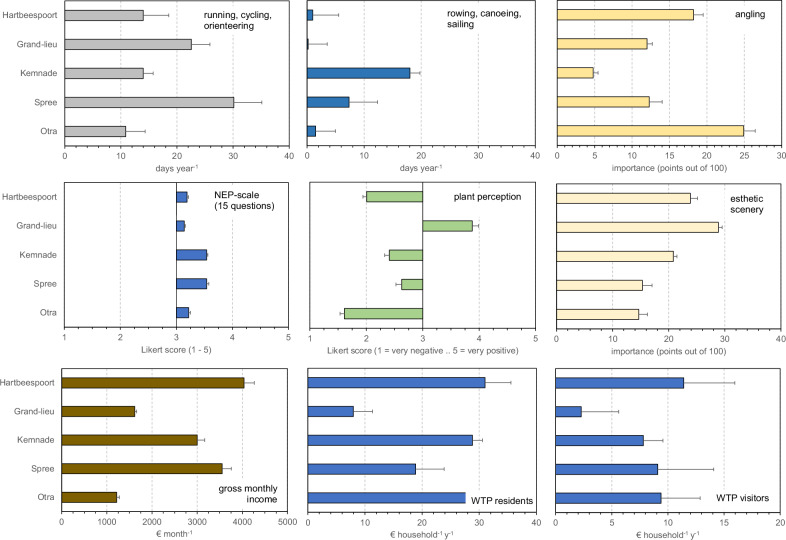
Among-site comparison of a selection of respondent characteristics pooled over visitors and residents. Presented are means plus 1 standard error, replication is variable. One-way ANOVAs for the nine presented plots had a p ≤ 0.001. (see Tables 2 and 3 for outcomes of more extensive analyses of variance)

**Fig. 4:**
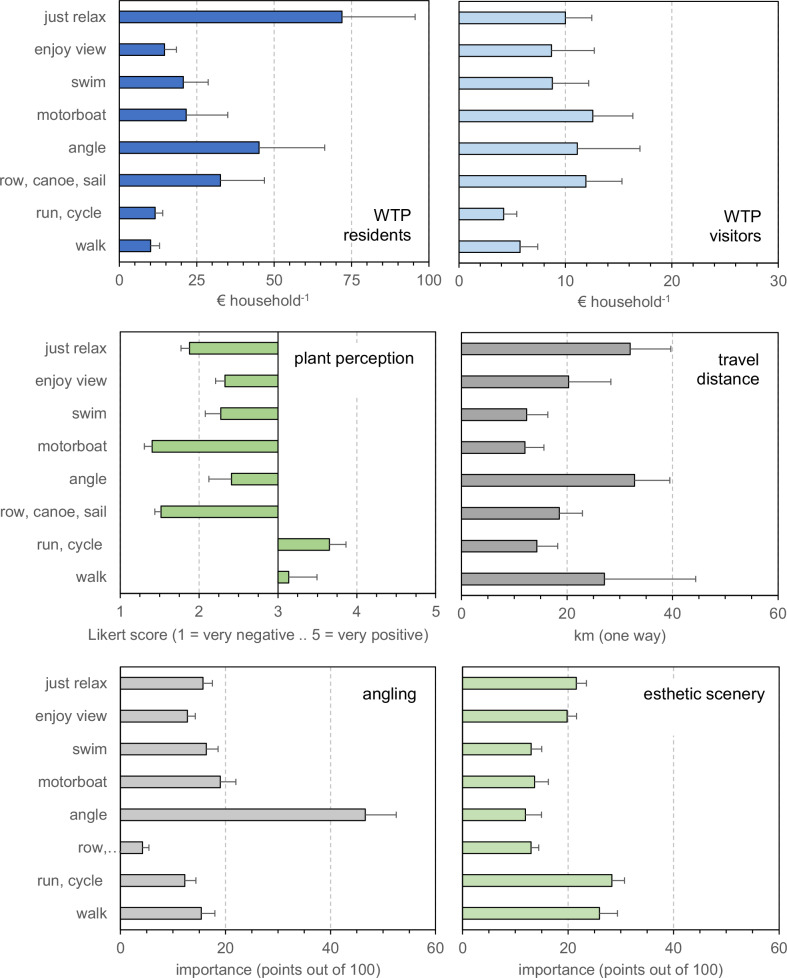
Comparison of selected respondent characteristics among ‘dedicated’ user categories of these 8 types of activity (see methods and Table 4 for the categorization procedure). Presented are means plus 1 standard error. Levels of significance of one-way ANOVAs were, respectively: WTP residents: 0.025, WTP visitors: 0.245, perception plants: <0.001, travel distance: <0.001, pts angling: <0.001, pts esthetic scenery: <0.001. The bottom two panels show the importance attached to angling (left) or esthetic scenery (right). Note that one-way ANOVAs pool possible effects of any other potentially explanatory variables (see Table 4)

In our exploratory two-way ANOVAs (Table 2), the differences among sites were most often significant (35 out of 39 tests), whereas this was less often the case for the difference between visitor and resident, and the interaction between the two factors (respectively 25 and 26 times). This implies that site is most often the most important factor explaining the observed patterns. Indeed, almost all one-way ANOVAs across sites were significant (Fig. 3 shows only a selection of all possible contrasts in Table 2). The quantified variables differed considerably in their among-site patterns (Fig. 3). Contrasting patterns among sites, for example, were present in types of activity (running and cycling most often done along the Spree and Lake Grand-Lieu, rowing and particularly sailing in Lake Kemnade) and the perception of the target plants (not considered very negative at Grand-Lieu, where the respondents also attached most importance to the esthetic scenery). We observed little difference in overall environmental mindedness as expressed with the NEP-scale (differences were significant but very small, only 7% between the highest and lowest mean scores). The reported monthly income among respondents did not reflect a pattern in national GDP, suggesting that people from different societal strata are the main recreating users of our study sites, but this is not related clearly to any activity type (like angling or boating). Then, the reported actual time spent on active watersports was fairly limited with the highest mean of 18 days per year (sum of rowing, sailing and canoeing) for the German Lake Kemnade. The mean number of days that respondents report swimming was highest in the Spree (17 ± 3). Days spent angling were highest for the Otra and Hartbeespoort Dam, but in the same range (around 20 days) as the maxima for swimming or active watersports (Fig. 3). When a range of covariates was included with site to explain variation in WTP for residents and visitors separately (Table 3), we found significant effects of gender (both residents and visitors) and higher education (residents), and the three two-way interactions (residents), but no effects of perception of the plants, environmental orientation (NEP-scale), age or travel distance. However, a similar analysis comparing groups of ‘dedicated’ respondents (Table 4), showed a different pattern, here the perception of plants had a significant effect on WTP for both residents and visitors. Among visitors these different user categories did not differ significantly (Table 4). With other words, it matters what you do whether you perceive these plants as a nuisance. The differences in WTP among ‘dedicated’ users correspond with differences in reported importance among categories (Fig. 4 bottom two plots): those using boats had a more negative perception of water plants than those that used the area for running or walking. Anglers found angling far more important than respondents that were dedicated to other uses, and the contrast was most striking with those engaged in rowing, canoeing or sailing. Particularly runners and walkers attached high importance to the esthetic scenery (Fig. 4).

**Table 3:** Analyses of co-variance of the dependence of willingness-to-pay for aquatic plant removal across five sites and a range of potentially explanatory variables.

Variable	Residents		Visitors	
	% model variance	p	% model variance	p
Age	1	0.085	1	0.111
NEP score	0	0.239	0	0.382
Travel distance	0	0.898	0	0.478
Perception of plants	0	0.224	1	0.103
Site	3	*0.016	3	*0.040
Gender	2	*<0.001	1	*0.039
Higher education	1	*0.009	1	0.075
Site × gender	3	*0.003	2	0.152
Site × higher education	4	*0.002	2	0.110
Gender × higher education	1	*0.025	1	0.167

Presented are the percentage of model variance explained by a variable and its level of significance (* < 0.05). Error degrees of freedom were 409 for residents and 348 for visitors. The three-way interaction was suppressed

**Table 4:** Analyses of covariance of the willingness-to-pay for aquatic plant removal among 8 different categories of ‘dedicated’ users: those engaged intensively in walking, running or cycling, rowing or canoeing or sailing, angling, using a motorboat, swimming, enjoying the view, or just relaxing.

Variable	Residents		Visitors	
	% model variance	p	% model variance	p
Age	0	0.215	0	0.295
NEP score	0	0.475	0	0.065
Travel distance	0	0.682	0	0.545
Perception of plants	1	0.051	2	*0.010
User category	6	*<0.001	2	0.648
Gender	1	0.135	0	0.510
Higher education	0	0.506	0	0.825
User category x gender	5	*0.002	1	0.843
User category x higher education	5	*0.001	1	0.971
Gender x higher education	1	0.084	1	0.071

Respondents were allocated to a category if they spend more or equal to the average number of days on that activity. Error degrees of freedom were 397 and 336. The three-way interaction was suppressed

### Underlying socio-cultural and economic drivers in willingness-to-pay

**Fig. 5:**
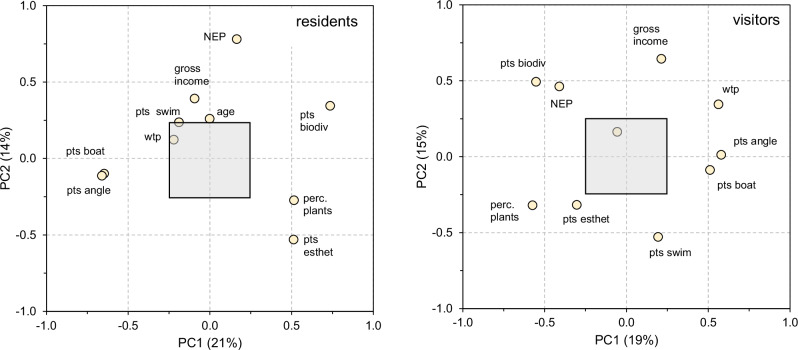
Covariance patterns of response variables and willingness to pay for aquatic plant removal for residents (*N* = 495) and visitors (*N* = 230). The number of variables included is a compromise between maximizing the number of respondents and the variation among user categories. Presented are the correlation coefficients of the original variables with the first (horizontal axis) and second principal component (vertical axis). the transparent quadrat shades the area with correlations <0.25, which are not considered to be significant. PC1 explains 21% for residents and 19% for visitors. For PC2 this is 13% and 16%, respectively. Abbreviations (see also Table 2): gross income = gross monthly income, ppp-corrected, in euros, NEP = New Environmental Paradigm score, perc plants = perception of plants (high is positive), pts angle = points out of 100 given to angling, pts biodiv = points to biodiversity conservation, pts boat = points to boating, pts esthet = points to esthetic scenery, pts swim = points to swimming, travdist = travel distance (km, one way, only included for visitors), wtp = willingness to pay for plant removal level 2 (cf Figs. 1 and 2 and Table 2)

Patterns of covariance of WTP with respondent traits were similar for residents and visitors (Fig. 5). The clearest similarity is a correlation with gross income (positive) and perception of the plants (negative), which is confirmed by a multiple regression of WTP with the same variables (gross income and perception significant at p < 0.01 or less, analyses not shown). The covariance patterns suggest that importance attached to boating and angling is at opposite ends with points given for esthetic scenery and biodiversity conservation, a contrast grasped by PC1 for residents, and the pattern is most clearly visible for visitors (Fig. 5). Visitors and residents differ in how close WTP is corelated to PC1, and the position of the NEP scores, otherwise the two plots are mainly just mirrored across the vertical. Gross monthly income is the strongest underlying factor for PC2 (Fig. 5). So, importantly, WTP for removal at level 2 increased with perceived nuisance among both residents and visitors (Fig. 5), but neither category expressed an increased WTP for increased removal intensity (Fig. 2). Also, pooled across the different sites, gross monthly income of individual respondents was a significant driver of WTP.

## Discussion

As an answer to our first question, our data suggest that respondents were not necessarily willing to pay more for mechanical removal of aquatic plants from lakes and rivers. We observed a significant but limited increase in WTP for more intensive plant removal in only two of the five sites. Not unexpectedly, both respondent characteristics and WTP for plant removal showed differences among the five sites. Differences between residents and visitors were less often significant than those between sites, but WTP of residents was higher than that of visitors. For one of our sites, the Otra, a previous assessment of the WTP for plant removal has been made by Navrud (2015), who used a benefit transfer to arrive at an estimate of ~130€ y^−1^ per local household at 2014 price levels. This is in the same order of magnitude but about 3–4 times higher than our estimates. The most likely reason is his use of benefit transfer from a different study (Barton et al. 2009), where WTP for a serious improvement of water quality was assessed in a lake with publicly well-known problems with cyanobacterial blooms. We assume that WTP for a significant water quality improvement is higher than for plant removal in a river with otherwise excellent water quality. This is in line with the hierarchy of water quality improvements (the so-called ‘water quality ladder’) suggested by e.g. Hanley and Black (2006) and Bateman et al. (2011).

Differences in WTP among recreative user categories were significant, but only among residents (Table 4), and the perception of the plants played an additional role as it was also significant. Overall, we found that the perception of the water plants was more positive among frequent (‘dedicated’) runners and walkers than among all the other categories, and their WTP for removal was comparatively low. Due to the design of our study, our observations do not allow the estimation of a specific nuisance threshold as suggested by Verhofstad and Bakker (2019), but our estimates of WTP and plant perception do not give unequivocal support for their assumption that such a threshold would be lowest for swimmers: dedicated anglers had a higher WTP than swimmers or boaters, and dedicated boaters reported the most negative plant perception. Regarding WTP, the group of residents that were mainly engaged in ‘just relaxing’ had the highest WTP, followed by anglers and boaters (Fig. 4). This is not supporting the suggestion of Beardmore (2015) that consumptive users would have a higher WTP than non-consumptive ones. We found a negative relation between WTP and the NEP-score in our PCA for visitors but not for residents. For the former the NEP score covaried closely with points given for biodiversity, whereas for the latter it was closer to income. The NEP scores showed no difference among user categories or sites in our ANCOVAs (Tables 3 and 4). Runners and walkers were the categories attaching most importance to esthetic scenery of the site.

Underlying socio-economic drivers for the observed variation in WTP were similar among residents and visitors: gross monthly income and perception of the plants both explained a significant proportion of the variation in WTP, particularly for boaters and anglers, whereas other factors covaried with these two or were of little importance. The two principal components explaining most of the covariance pattern can be interpreted as one dimension separating the importance attributed to boating versus that of biodiversity, or esthetic scenery, and the second dimension representing income. This overall pattern is apparent over and above the considerable differences observed among sites. Based on the proximity in the PCA plot of importance attached to boating and to angling, we suggest that the first PCA-dimension corresponds with the finding of Beardmore (2015), who found a distinct difference between anglers and sight-seeing visitors. We found little evidence for effects of employment, or a rural versus urban living environment in our analyses. Higher education and gender were significant in our ANCOVA comparing sites, but not in the one comparing ‘dedicated’ user categories (Tables 3 and 4), supporting the suggestion that the site contrast is strong and is reflected in many different respondent characteristics, prevalence of recreative activities as well as WTP for plant removal (cf the significance patterns in Table 2). Our analysis of ‘dedicated’ user categories did not confirm the observation by Arlinghaus (2006, 2021) that angling is a male-dominated activity, possibly because this activity is less male-dominated in the two sites where angling was most considered most important, i.e. South Africa and Norway. Aas (1996), indeed reported the absence of a male bias for Norway, notably among occasional anglers.

A few striking differences among the sites deserve discussion. First, the striking difference in how many days the respondents report to spend on active water sports does not correspond with their perception of the water plants (Fig. 3). Our PCAs suggest that it is rather angling and the use of motorboats that corresponds with a WTP for removal. Second, contrary to the other sites, respondents in Lake Grand-Lieu do not report a negative perception of the local mass development of *Ludwigia* spec., and also report a substantially lower WTP for its removal, than what is observed at the other sites. One can only speculate whether this is due to the fact that *Ludwigia* has such conspicuous flowers, or whether the respondents indeed do not experience the plant as a serious problem, although its invasive character is well publicized (Geffray and Gerard 2018). A plausible reason based on our observations may be that respondents at Grand-Lieu are more engaged in running and cycling than the average, and less in rowing, boating and angling, whilst they attach more importance to the esthetics of the scenery. In Hartbeespoort Dam, the WTP among residents showed a very distinct pattern unlike that in all other sites. Here, the WTP was highest for the current removal intensity, to decline rapidly at ‘level 2’ (Fig. 2). We suggest it is likely that local respondents are well aware of the likelihood of intensive cyanobacterial blooms after large-scale water hyacinth removal, which may cause anoxia and fish kills (Van Ginkel, 2011). This is illustrated by the following response (literal citation) to one of our open-ended questions: “Water hyacinth in the HBPD is a necessary evil in the fight against extreme algae (in extreme cases blue toxic) which forms in the absence of hyacinth, as a result of the very toxic and raw affluent and waste that comes down mainly the Crocodile river”.

The low WTP for increased water plant removal is an important message for water managers considering to generate additional funds to cover costs. Also, about half of the respondents (Table 2) expressed that they were not willing to pay at all, so they explicitly filled out the value ‘zero’ (and these are included in our calculations of mean WTP). These respondents gave several, sometimes overlapping reasons ranging from a limited trust in authorities, to the opinion that a tax increase is unrealistic or the observation that plant removal is not dealing with the ultimate cause. In addition, the issue of hypothetical bias or the difference between stated and revealed WTP requires consideration. Stated WTP can be a factor 2–3 higher than actual, revealed WTP (Loomis 2011), but we have no means to estimate the magnitude of this potential bias here. the values observed here are fairly low (medians across sites range from 3–6 hh^−1^ in household tax for residents and 1–5 hh^−1^ in tourist tax for visitors), but still any estimate of a population-wide total should be done with caution.

Methodological concerns can be raised over several issues: (1) The complexity of the survey may have led to fatigue, unreflected outlier responses or premature abandonment among respondents; (2) Willingness to participate in our survey may have led to an undesired and biased selection of respondents from the general population; (3) The selection of the subcategory ‘dedicated’ users may have caused bias. Regarding the first issue, we observed and removed only few obvious protest responses (see Thiemer et al. 2023). Then, over 1000 of the 1780 respondents completed the WTP question in the survey and these respondents still reflected a wide range of income and age, as well as a moderate NEP-score similar in range to e.g. Immerzeel et al. (2022) or Hawcroft, Milfont (2010). Thus, with statistical designs allowing between 336 and 409 error degrees of freedom (Tables 3 and 4), we conclude that respondent bias is not likely to play an important role. The second issue is a principal one, which is the consequence of our approach where we sought up our respondents in situ rather than making use of panel data. We are convinced that our study benefitted from surveying opinions and value statements among different groups of real-world users that are confronted with mass development of macrophytes in their lake or river, rather than having to depend on a set of panel answers from respondents that have a weak relation with the site, if any. The third point is our selection of respondents specifically ‘dedicated’ to a particular activity, where we selected those reporting to spend more than or equal to the average number of days on that specific activity. This selection criterion rather than a reported membership of e.g. a sailing club in our view is more appropriate as it assumes actual use. It is purposefully selective and we can derive specific needs of particular groups from it. At the same time, the selection procedure led to variable proportions of the full sample included, for example for the WTP of residents this was 49% and for visitors this was 67%. Possibly counterintuitive this selection also revealed that particularly the ‘dedicated’ user category reporting to ‘just relax’ showed the highest WTP, both among residents and visitors. This category represents a measurable proportion of our ‘dedicated’ users: 15 and 27% of residents and visitors, respectively. For comparison, ‘dedicated’ anglers and motorboaters together form on average 12 and 20% whereas those that row, canoe or sail represent 7 and 31% of the intensive users, when pooled across the five sites.

In conclusion, we found that (1) an increase in WTP for increased water plant removal was absent in most sites; (2) indeed user groups appear to differ in their WTP for plant removal, but the pattern is not straightforward; (3) pooled across sites, the two main underlying drivers of WTP for water plant removal were income and engagement with angling and boating, and these did not differ strongly between visitors and residents; (4) differences among sites were distinct and related to different mixtures of activity patterns linked to differences in the perception of the plants. The case-specificity of the five sites was important in explaining variation in current WTP for water plant removal. Water managers should thus be aware of the limited WTP for increased removal, and tailor management schemes to the prevailing activities, this may involve targeted partial removal in most heavily used areas and directed spatial separation of different uses.

## Data Availability

Survey data are deposited in DATAVERSE.NO at 10.18710/FUWNJL according to the contract with the Norwegian Research Council.

## References

[CR1] Aas Ø (1996) Recreational fishing in Norway from 1970 to 1993: trends and geographical variation. Fish Manag Ecol 3:107–118

[CR2] Arlinghaus R (2006) Understanding recreational angling participation in Germany: preparing for demographic change. Hum Dim Wildl 11:229–240

[CR3] Arlinghaus R, Aas Ø, Alós J, Arismendi I, Bower S, Carle S, Czarkowski T, Freire KMF, Hu J, Hunt LM, Lyach R, Kapusta A, Salmi P, Schwab A, Tsuboi J, Trella M, McPhee M, Potts W, Wołos A, Yang Z (2021) Global participation in and public attitudes toward recreational fishing: international perspectives and developments. Rev Fish Sci Aquacult 29:58–95

[CR4] Arp RS, Fraser GCG, Hill MP (2017) Quantifying the economic water savings benefit of water hyacinth (*Eichhornia crassipes*) control in the Vaalharts irrigation scheme. Water SA 43:58–66

[CR5] Barton D, Navrud S, Lande N, Bugge Mills A (2009) Assessing Economic Benefits of Good Ecological Status in Lakes under the EU Water Framework Directive. Case Study Report from the EU-project “Aquamoney”. Norwegian Institute for Water Research (NIVA), NIVA Report 5732-2009, 109 pp.

[CR6] Bateman IJ, Brouwer RJ, Ferrini S, Schaafsma M, Barton DN, Dubgaard A, Hasler B, Hime S, Liekens I, Navrud S, De Nocker L, Sceponaviciute R, Semeniene D (2011) Making benefit transfers work: deriving and testing principles for value transfers for similar and dissimilar sites using a case study of the non-market benefits of water quality improvements across Europe. Env Res Econ 50:365–387

[CR7] Beardmore B (2015) Boater perceptions of environmental issues affecting lakes in northern Wisconsin. J Am Wat Res Assoc 51:537–549

[CR8] Bergstrom JC, Teasley RJ, Cordell HK, Souter R, English DBK (1996) Effects of reservoir aquatic plant management on recreational expenditures and regional economic activity. J Agric Appl Econ 28:409–422

[CR9] Boerema A, Schoelynck J, Bal K, Vrebos D, Jacobs S, Staes J, Meire P (2014) Economic valuation of ecosystem services, a case study for aquatic vegetation removal in the Nete catchment (Belgium). Ecosyst Serv 7:46–56

[CR10] Clayton JS (1996) Aquatic weeds and their control in New Zealand lakes. Lake Reserv Manag 12:477–486

[CR11] Dehez J (2023) An investigation of outdoor recreational users’ willingness to participate in aquatic invasive plant control. Biol Conserv 277:109830

[CR12] Dunlap RE, Heffernan RB (1975) Outdoor recreation and environmental concern: an empirical examination. Rural Socio 40:18–30

[CR13] Dunlap RE, Van Liere KD, Mertig AG, Jones RE (2000) New trends in measuring environmental attitudes: measuring endorsement of the new ecological paradigm: a revised NEP scale. J Socio 56:425–442

[CR14] Geffray O, Gerard B (2018) Plantes aquatiques exotiques invasives. Report Federation de Loire Atlantique pour la peche et la protection du milieu aquatique DPPMA44, Nantes.

[CR16] Hadwen WL, Arthington AH, Mosisch TD (2003) The impact of tourism on dune lakes on Fraser Island, Australia. Lakes Reserv: Res Manag 8:15–26

[CR17] Hanley N, Black AR (2006) Cost-benefit analysis and the Water Framework Directive in Scotland. Integr Env Assess Manag 2:156–165 16646384

[CR18] Hawcroft LJ, Milfont TL (2010) The use (and abuse) of the new environmental paradigm scale over the last 30 years: A meta-analysis. J Environ Psychol 30:143–158

[CR20] Hill M, Coetzee J (2017) The biological control of aquatic weeds in South Africa: Current status and future challenges. Bothalia 47:a2152

[CR21] Hilt S, Gross EM, Hupfer M, Morscheid H, Mählmann J, Melzer A, Poltz J, Sandrock S, Scharf EM, Schneider S, Van de Weyer K (2006) Restoration of submerged vegetation in shallow eutrophic lakes–A guideline and state of the art in Germany. Limnologica 36:155–171

[CR22] Hussner A, Stiers I, Verhofstad MJJM, Bakker ES, Grutters BMC, Haury J, Van Valkenburg JLCH, Brundu G, Newman J, Clayton JS, Anderson LWJ, Hofstra D (2017) Management and control methods of invasive alien freshwater aquatic plants: A review. Aquat Bot 136:112–137

[CR23] Immerzeel B, Vermaat JE, Juutinen A, Pouta E, Artell J (2022) Why we appreciate Nordic catchments and how the bioeconomy might change that: results from a discrete choice experiment. Land Use Pol 113:105909

[CR24] Johnston RJ, Boyle KJ, Adamowicz W, Bennett J, Brouwer R, Cameron TA, Hanemann WM, Hanley N, Ryan M, Scarpa R, Tourangeau R, Vossler CA (2017) Contemporary guidance for stated preference studies. J Assoc Environ Resour Econ 4:319–405

[CR25] Loomis J (2011) What’s to know about hypothetical bias in stated preference valuation studies? J Econ Surv 25:363–370

[CR26] Misteli B, Pannard A, Aasland E, Harpenslager SF, Motitsoe S, Thiemer K, Llopis S, Coetzee J, Hilt S, Köhler J, Schneider SC, Piscart C, Thiebaut G (2023) Short-term effects of macrophyte removal on aquatic biodiversity in rivers and lakes. J Env Manag 325:116442 10.1016/j.jenvman.2022.116442 36244282

[CR27] Murphy KJ, Eaton JW (1983) Effects of pleasure boat traffic on macrophyte growth in canals. J Appl Ecol 20:713–729

[CR28] Navrud S (2015) Sammfunnsøkonomisk nytteveri av tiltak mot krypsiv. VISTA ANALYSE report 2015/5. Oslo

[CR29] Navarro DJ Foxcroft DR (2022) Learning statistics with jamovi: a tutorial for psychology students and other beginners. (Version 0.75). 10.24384/hgc3-7p1

[CR30] Nikolaus K, Schafft M, Maday A, Klefoth T, Wolter C, Arlinghaus R (2020) Status of aquatic and riparian biodiversity in artificial lake ecosystems with and without management for recreational fisheries: Implications for conservation. Aquat Conserv: Mar Freshwat Ecosys 31:153–172

[CR31] Ostendorp W, Gretler T, Mainberger M, Peintinger M, Schmieder K (2009) Effects of mooring management on submerged vegetation, sediments and macro-invertebrates in Lake Constance, Germany. Wetl Ecol Manag 17:525–541

[CR32] Pieterse AH, Murphy KJ (1990) Aquatic weeds: the ecology and management of nuisance aquatic vegetation. Oxford University Press, Oxford, New York.

[CR33] Podraza P, Brinkmann T, Evers P, Von Felde D, Frost U, Klopp R, Knotte H, Kühlmann M, Kuk M, Lipka P, Nusch EA, Stenert M, Wessel M, Van de Weyer K (2008) F & E- Vorhaben im Auftrag des Ministeriums für Umwelt und Naturschutz, Landwirtschaft und Verbraucherschutz des Landes NRW (MUNLV), Aktenzeichen: 54.173/25-5230), Report Ruhrverband

[CR34] Schafft M, Wegner B, Meyer N, Wolter C, Arlinghaus R (2021) Ecological impacts of water-based recreational activities on freshwater ecosystems: a global meta-analysis. Proc R Soc B 288:20211623 10.1098/rspb.2021.1623 PMC8456150 34547908

[CR35] Thiemer K, Schneider SC, Demars BOL (2021) Mechanical removal of macrophytes in freshwater ecosystems: Implications for ecosystem structure and function. Sci Tot Env 782:146671 10.1016/j.scitotenv.2021.146671 33838383

[CR36] Thiemer K (2022) Mass development of macrophytes – causes and consequences of macrophyte removal for ecosystem structure, function and services. PhD-thesis Norwegian University of Life Sciences, Ås, Norway.

[CR37] Thiemer K, Immerzeel B, Schneider S, Sebolwa K, Baldo M, Thiebaut G, Hilt S, Köhler J, Harpenslager SF, Vermaat JE (2023) Underlying drivers for perceived nuisance growth of aquatic plants. Env Manag 71:1024–1036 10.1007/s00267-022-01781-x PMC9832253 36627533

[CR38] Van Ginkel CE (2011) Eutrophication: present reality and future challenges for South Africa. Water SA 37:693–701

[CR39] Venohr M, Langhans SD, Peters O, Hölker F, Arlinghaus R (2018) The underestimated dynamics and impacts of water-based recreational activities on freshwater ecosystems. Env Rev 26:199–213

[CR40] Verhofstad MJJM, Bakker ES (2019) Classifying nuisance submerged vegetation depending on ecosystem services. Limnology 20:55–68

[CR41] Vermaat JE, Thiemer K, Immerzeel B, Schneider SC, Sebola K, Petruzzella A, Coetzee J, Baldo M, Misteli B, Thiebaut G, Hilt S, Köhler J, Harpenslager SH (2024) Effects of managing nuisance abundance of aquatic plants on a suite of ecosystem services. J Appl Ecol 61:76–89

[CR42] Villamagna AM, Murphy BR (2010) Ecological and socio-economic impacts of invasive water hyacinth (*Eichhornia crassipes*): a review. Freshwat Biol 55:282–298

[CR43] Wegner B, Meyer N, Wolter C (2023) Paddling impacts on aquatic macrophytes in inland waterways. Jr Nat Conserv 72:126331

